# Silver Oxide Coatings with High Silver-Ion Elution Rates and Characterization of Bactericidal Activity

**DOI:** 10.3390/molecules22091487

**Published:** 2017-09-07

**Authors:** Sarah S. Goderecci, Eric Kaiser, Michael Yanakas, Zachary Norris, Jeffrey Scaturro, Robert Oszust, Clarence D. Medina, Fallon Waechter, Min Heon, Robert R. Krchnavek, Lei Yu, Samuel E. Lofland, Renee M. Demarest, Gregory A. Caputo, Jeffrey D. Hettinger

**Affiliations:** 1 Department of Chemistry and Biochemistry, Rowan University, Glassboro, NJ 08028, USA; godere61@students.rowan.edu (S.S.G.); clarence.d.medina@dupont.com (C.D.M.); Fstrope6@gmail.com (F.W.); yu@rowan.edu (L.Y.); 2 Department of Physics and Astronomy, Rowan University, Glassboro, NJ 08028, USA; erk214@lehigh.edu (E.K.); yanaka73@students.rowan.edu (M.Y.); norris48@students.rowan.edu (Z.N.); Scaturro@bu.edu (J.S.); oszust01@students.rowan.edu (R.O.); lofland@rowan.edu (S.E.L.); 3 Department of Materials Science and Engineering, Drexel University, Philadelphia, PA 19104, USA; minheon@gmail.com; 4 Department of Electrical and Computer Engineering, Rowan University, Glassboro, NJ 08028, USA; krchnavek@rowan.edu; 5 Department of Molecular Biology, Rowan University, Stratford, NJ 08084, USA; demarest@rowan.edu; 6 Department of Molecular and Cellular Biosciences, Rowan University, Glassboro, NJ 08028, USA

**Keywords:** bactericidal coatings, antibacterial, silver oxide, reactive sputtering, thin film coatings

## Abstract

This paper reports the synthesis and characterization of silver oxide films for use as bactericidal coatings. Synthesis parameters, dissolution/elution rate, and bactericidal efficacy are reported. Synthesis conditions were developed to create AgO, Ag_2_O, or mixtures of AgO and Ag_2_O on surfaces by reactive magnetron sputtering. The coatings demonstrate strong adhesion to many substrate materials and impede the growth of all bacterial strains tested. The coatings are effective in killing *Escherichia coli* and *Staphylococcus aureus*, demonstrating a clear zone-of-inhibition against bacteria growing on solid media and the ability to rapidly inhibit bacterial growth in planktonic culture. Additionally, the coatings exhibit very high elution of silver ions under conditions that mimic dynamic fluid flow ranging between 0.003 and 0.07 ppm/min depending on the media conditions. The elution of silver ions from the AgO/Ag_2_O surfaces was directly impacted by the complexity of the elution media, with a reduction in elution rate when examined in complex cell culture media. Both *E. coli* and *S. aureus* were shown to bind ~1 ppm Ag^+^/mL culture. The elution of Ag^+^ resulted in no increases in mammalian cell apoptosis after 24 h exposure compared to control, but apoptotic cells increased to ~35% by 48 and 72 h of exposure. Taken together, the AgO/Ag_2_O coatings described are effective in eliciting antibacterial activity and have potential for application on a wide variety of surfaces and devices.

## 1. Introduction

Antimicrobial resistance has proven to be an obstacle of overwhelming importance to both the medical and scientific communities following the introduction of antibiotics in the early 20th century [1]. The ability of many bacteria to adapt to changing environmental conditions can be at least partly a result of their ability to overcome pharmaceutical approaches intended to eradicate them. Regular and improper antibiotic use is partially responsible for providing advantages to mutant bacterial strains; patients infected with those mutant strains have proven far more costly to treat and often require longer hospital stays in comparison to patients with strains that are susceptible to traditional, small-molecule antibiotic treatments [1,2]. In addition to acquired resistance, intrinsic resistance is another factor in the resistance cascade: the low membrane permeability of certain bacterial strains makes antibiotic penetration of the membrane nearly impossible [1]. Current projections estimate that if there are no successful efforts to combat the spread of resistance, the number of deaths could increase from the current reported annual total of 700,000 to as many as 10 million by the year 2050 [3].

Preventing infections has become a goal of both the scientific and medical communities in an attempt to reduce antibiotic usage and stop the spread and/or development of resistant strains. Infection control has become a significant concern in many areas of medicine, but is especially critical in orthopedic surgery due to the invasive measures that must be taken when infections develop post-surgery and are extremely difficult to treat with antibiotics alone. The restricted access around surgical implants results in the need for stronger and longer antibiotic courses and increased potential for implant retrieval, debridement, and device replacement. Additional surgeries result in increases in patient stress and prolonged hospital stay [4]. Some studies estimate that 2.5% of primary hip and knee arthroplasties and up to 20% of revision arthroplasties develop a periprosthetic joint infection, with the mortality rate for these infections at nearly 2.5% [5].

Silver has been used for thousands of years in different civilizations for numerous applications, including food and water purification, ulcer treatments, promoting wound healing, as well as prevention of surgical infections [6]. Antimicrobial studies using silver compounds show its efficacy against a wide range of bacterial species, including *Bacillus subtilis*, *Escherichia coli*, *Pseudomonas aeruginosa*, *Proteus vulgaris*, and *Staphylococcus aureus* [7,8,9,10]. The exact antimicrobial mechanism of action of silver is not fully elucidated, but the ability of silver to act against multiple bacterial species suggests that silver interacts with multiple bacterial target sites, most readily with the thiol groups of cysteine residues [11]. This is consistent with the high abundance of thiol groups in bacterial cell membranes and silver exhibiting a broad-spectrum antimicrobial activity profile. At the bacterial cell membrane, ionic silver has been shown to inhibit the proton motive force (PMF), the respiratory electron transport chain, and affect membrane permeability, all of which can result in cell death [12,13,14]. Although different forms of silver are available, the ones most readily available fall into three categories: elemental silver, inorganic silver complexes, and organic silver complexes [14].

Many varieties of silver materials are used in both healthcare and industry, yet the release of silver ions from the complex is what ultimately determines the compound’s antimicrobial efficacy [15]. The primary drawback of pure silver is the negligible aqueous solubility, which limits the ability to act as an antimicrobial over meaningful distances from metallic surfaces. Considering that the silver ion (Ag^+^-ion) is the most likely antimicrobial form, efficient solubilization in the environment is necessary for activity beyond the surface [16]. Silver nanoparticles (Ag-NP) as antimicrobial coatings requires the particles to be embedded in a matrix of another material to adhere to implant surfaces, creating convoluted paths which suppress the overall elution rate. The rate of Ag^+^-ion elution from Ag-NP also may not be sufficient to significantly impact the growth of large numbers of bacteria in the complex environment of the human body. Silver oxide has higher solubility in water compared to pure metallic Ag, and has been shown to generate enough Ag^+^-ions to affect antimicrobial activity in several applications [17,18].

Armed with the knowledge that the Ag^+^-ion has broad-spectrum bactericidal properties, and also recognizing the shortcomings of metallic Ag- and Ag-NP-containing coatings, this work focuses on developing Ag-based coatings with high dissolution rates in comparison to various forms of metallic Ag, yielding meaningful levels of Ag^+^-ions in solution to inhibit bacterial growth. Though some work has been reported that synthesizes silver oxide materials for semiconductor applications [19,20], absent from the literature is a systematic investigation of the materials’ characterization under relevant conditions for bactericidal activity. Compounds in the silver oxide family can be deposited reactively at room temperature and have orders-of-magnitude higher dissolution rates than that of pure metallic Ag. The coatings were analyzed for molecular composition, surface morphology, and the elution properties of Ag^+^ ions in a variety of aqueous solutions. The antibacterial activity against model Gram positive and Gram negative bacterial strains is presented. The results indicate that the solubility of silver oxide coatings is directly linked to the antibacterial activity and that these materials are promising for future development as device coatings to mitigate bacterial infections.

## 2. Results

### 2.1. Oxygen Flow Dependence of Oxide Formed

**Figure 1:**
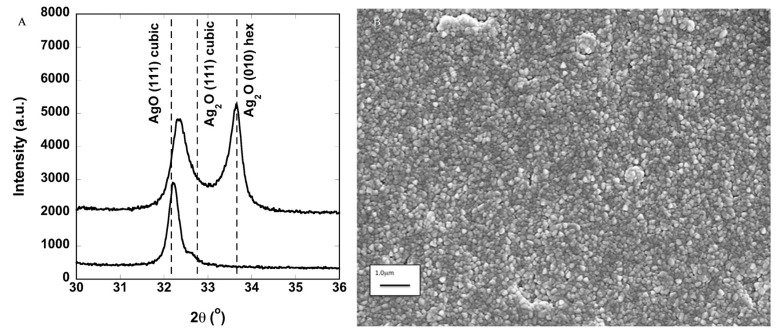
(**A**) Plot of the X-ray diffraction intensity versus 2θ showing single phase cubic AgO and mixed phase AgO and Ag_2_O deposited at lower oxygen partial pressure; (**B**) Scanning electron micrograph showing the typical surface microstructure of the silver oxide deposited at room temperature. The microstructure can be impacted by deposition pressure, deposition power, oxygen partial pressure, and coating thickness.

Silver oxide films were deposited for 30 min on polished *C*-axis sapphire and other substrates by magnetron sputtering at a power of 100 W and a pressure of 25 mTorr. The XRD results of films grown at two different O_2_ partial pressures are shown in Figure 1A. Three phases of silver oxide could be expected to form at room temperature: Ag_2_O (hexagonal), Ag_2_O (cubic), and AgO (cubic). When the O_2_/Ar gas ratio is 2.5/60, Ag_2_O (hexagonal) and some cubic phase forms, as indicated by the position of the XRD peak. When the O_2_/Ar is 0.67, phase pure AgO (cubic) forms. Isolating AgO is the most straightforward, while the other phases tend to be mixed. Coatings deposited in this way are polycrystalline films. The XRD peak relative intensities do not match that expected for powders, suggesting that interactions with the substrate may cause some texturing. 

Scanning electron microscopy (SEM) was also performed on a representative coating deposited on a Ti-foil substrate, shown in Figure 1B. This image shows that the grains were fairly uniform in size at approximately 100 nm. The small grain size is typical for room temperature depositions. The small grains are also consistent with the relatively weak diffraction peaks observed.

**Figure 2:**
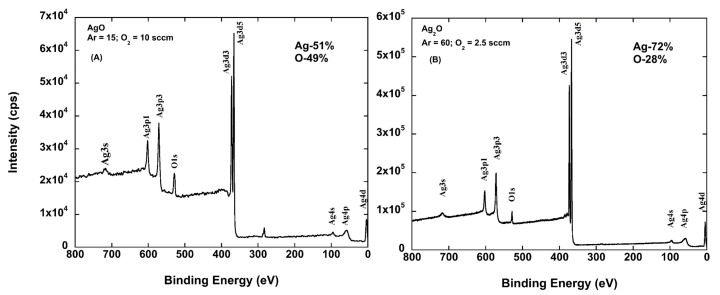
Plot of the X-ray photoelectron spectrum of samples deposited at room temperature at a power of 100 W and a pressure of 25 mTorr. (**A**) The spectrum for the sample shown as AgO in Figure 1A. Note that the atomic composition is nearly 1:1; (**B**) The spectrum for the sample deposited at lower partial pressure of O_2_ which results in mixed AgO and Ag_2_O. Note that the overall sample appears to be deficient in O for even Ag_2_O alone.

For the same films, XPS was performed. These results are shown in Figure 2A,B. The compositions are in general agreement with the XRD data. For the Ag_2_O sample, the XPS results indicate that there is an excess of Ag (higher than stoichiometric), although there are no additional diffraction peaks such as those associated with the formation of other phases or pure metallic Ag. Subsequently, the silver oxide films will be referenced as Ag*_x_*O, indicating the mix of AgO and Ag_2_O present in the films.

### 2.2. Adhesion of Ag_x_O Films

Adhesion testing performed by the ASTM D3359 cellophane tape test demonstrated that the as-deposited films were strongly adhered to the substrate with no need for a buffer layer. The substrates used included single crystal Al_2_O_3_, Ti, and the flexible polymer substrates. Even after creasing the flexible substrates, which resulted in fractures in the coatings, the adhesion test did not remove the coating, indicating that they are uniformly strong rather than at a few strong adhesion sites.

### 2.3. Ag Elution from Silver Oxide Films

**Figure 3:**
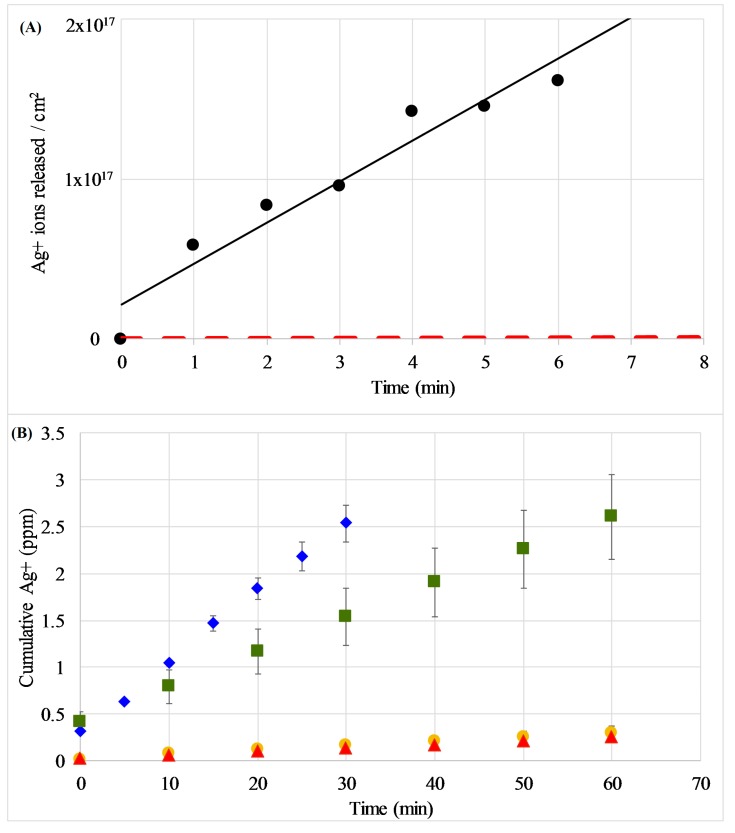
(**A**) Elution of Ag ions from Ag*_x_*O coatings (circles) in distilled water as measured by ICP-MS. Release profile is compared to estimated elution data from Ag nanoparticles (red dashed line near *X*-axis) from [21]; (**B**) Elution of Ag ions from Ag*_x_*O coatings in water (blue diamonds), PBS (green squares), LB growth medium (yellow circles) and DMEM growth medium (red triangles) as measured by ICP-MS. Data are averages with standard deviations of at least three replicates. In some cases, the error bars are smaller than the size of the symbol.

Silver oxide coatings were sputtered on Ti foils, and discs of 0.25″ diameter were punched from the foils to be placed in fixed volume deionized water baths and timed to test the Ag elution from the coating into the solution. The Ag^+^-ion concentrations resulting in the solutions were measured by inductively coupled plasma mass spectroscopy ICP-MS. The data plotted in Figure 3A (circles) represent the concentration of Ag^+^-ions measured in the solutions. These results are compared to the elution of Ag^+^-ions from silver nanoparticles shown as a red dashed line as measured by Kent et al. [21].

These measurements were extended to examine the effect of environment on the release rates, as the majority of previous reports have focused on the release profile in water, while in vitro cellular experiments take place in much more complex media. Figure 3B demonstrates the cumulative Ag release in water and various media types as measured by ICP-MS. The results show that the Ag^+^ elutes at a faster rate in water compared to the more complex media tested (LB used for bacterial growth experiments and DMEM used for mammalian cell growth conditions). Interestingly, the release rate of simple phosphate buffered saline (PBS) is slower than water but faster than the complex growth media. The overall trend of release rates is H_2_O > PBS > LB ≈ DMEM. The release rates calculated from these experiments and statistics are included in the Supplement (Table S1).

### 2.4. Antibacterial Activity

#### 2.4.1. Static Testing

**Figure 4:**
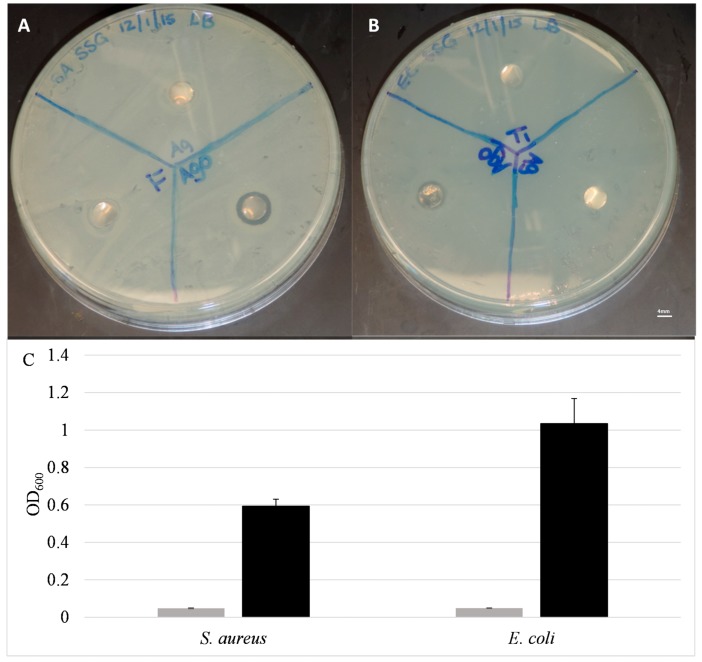
Antimicrobial activity of Ag*_x_*O coatings (**A**,**B**) Zone of inhibition assay. Images of LB plates seeded with (**A**) *Staphylococcus aureus* or (**B**) *Escherichia coli*. The Ag*_x_*O disc (labeled AgO on the plates) exhibited a zone of inhibition (ZOI) of (**A**) 9 mm for *S. aureus*; (**B**) 10 mm for *E. coli*. The diameter of the disc in each case is 6.5 mm (1/4”). No ZOIs were evident for Ag-coated or Ti control discs; (**C**) Overnight growth of *S. aureus* and *E. coli* in modified minimal inhibitory concentration (MIC) experiment. Gray bars represent cultures exposed to Ag*_x_*O-coated discs while black bars represent cultures exposed to uncoated Ti discs. Data are averages of at least three samples.

The ability of the coatings to release Ag^+^ into solution indicated that these coatings may be effective against bacteria that are not directly in contact with the surfaces. This is in contrast to previous findings with pure Ag coatings, which demonstrated resistance to bacterial colonization/adhesion, but did not provide any antibacterial activity beyond the surface [18]. The ability to exert antibacterial activity distal to the coated surface was first examined with a modified version of the Kirby–Bauer disc diffusion assay. Discs were laid onto LB-agar plates pre-seeded with *S. aureus* or *E. coli* (Figure 4A,B) and allowed to incubate overnight. The presence of a zone of inhibition (ZOI) indicates that the Ag^+^ ions from the coating eluted and inhibited bacterial growth with this zone. The Ti control disc and the Ag-coated discs both exhibited no measurable ZOI, indicating that the pure silver coatings were unable to elute and diffuse through the solid agar medium to any significant concentration able to prevent bacterial growth. In contrast, the Ag*_x_*O-coated discs clearly caused a ZOI, ranging from 0.8 to 1.0 cm in diameter. This inhibition of bacterial growth around the disc clearly indicates that Ag^+^ is eluting from the disc at high enough concentrations to inhibit bacterial growth on semi-porous solid surfaces.

A modified version of the traditional minimal inhibitory concentration (MIC) assay was employed to determine the ability of the coatings to inhibit bacterial growth in liquid culture. In this experiment, coated or uncoated Ti-discs were added to wells of a 96-well plate, and a known concentration of bacteria (5 × 10^5^–5 × 10^6^ CFU/mL) was then added. The optical density at 600 nm (OD_600_) of the cultures were measured after overnight incubation at 37 °C. In the cases of both *S. aureus* and *E. coli*, there was little or no turbidity observed in the cultures exposed to Ag*_x_*O-coated discs, indicating bacterial growth was inhibited. This is in contrast to the control samples containing uncoated Ti-discs, which exhibited high OD_600_ readings indicating robust bacterial growth (Figure 4C).

#### 2.4.2. Bacterial Growth Kinetics

**Figure 5:**
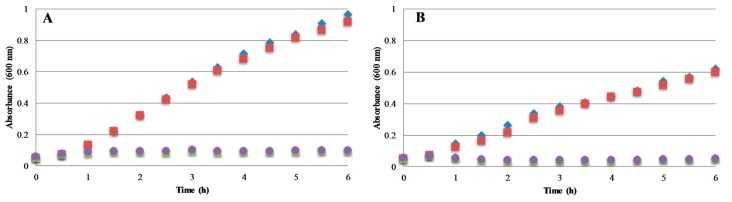
Antibacterial activity of Ag*_x_*O coatings in liquid culture. Absorbance (i.e., optical density at 600 nm (OD_600_)) is a measure of the number of bacterial cells in the solution. Ti or Ti discs coated with Ag*_x_*O were added to cultures of (**A**) *S. aureus* (starting density between 4 × 10^6^ and 4 × 10^7^ CFU/mL); or (**B**) *E. coli* (starting density ~3.73 × 10^7^ CFU/mL). Symbols are (green ▲, purple ●) for Ag*_x_*O-coated discs, (blue ♦) for untreated control, and (red ■) for uncoated Ti disc control. Experiments were performed at 37 °C with constant shaking of the cultures except during OD measurement.

Based on the previous findings of Ag*_x_*O coatings exhibiting antimicrobial activity in solution, measurements were extended to a more detailed analysis of coating properties on efficacy. Figure 5 shows the growth curves of the same standard model bacterial species in the presence of an uncoated Ti disc or a Ti disc coated with ~150 nm thick Ag*_x_*O film. In each case, the culture containing the Ti disc exhibited growth comparable to the control culture. In contrast, the cultures containing the Ag*_x_*O-coated discs showed significantly lower OD_600_ values for all bacteria tested. These curves indicate that the Ag*_x_*O coating is eluting from the surface of the disc into solution, inhibiting bacterial growth in all bacterial species. Additionally, this inhibition was almost immediate upon the addition of the Ag*_x_*O coated disc to the bacterial culture, indicating a rate of release high enough to very quickly reach a threshold concentration of Ag^+^-ion in the culture to inhibit growth.

#### 2.4.3. Bacterial Uptake of Ag

**Figure 6:**
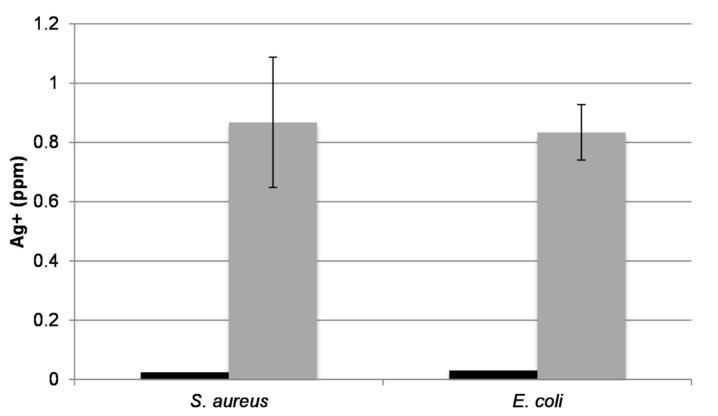
ICP-MS analysis of Ag^+^ from growth curve samples shown in Figure 5. Samples were removed at the end of the 6-h time course. Black bars represent samples taken from cultures treated with the uncoated Ti discs while the gray bars represent samples treated with Ag*_x_*O-coated Ti discs. Error bars represent ranges for Ag*_x_*O samples based on the replicate from each bacterial strain.

The results in the previous experiments show that the eluted Ag ions are the driving force behind the antimicrobial activity of the Ag*_x_*O coatings. These experiments were then extended to investigate the association of eluted Ag^+^-ion to bacteria in solution. Starting with cultures used in the growth curve experiments in Figure 5, a portion of the culture was pelleted, dissolved in 5% nitric acid, and subjected to ICP-MS analysis. In all cases, bacteria exposed to Ag*_x_*O-coated discs displayed significantly higher Ag content in the pelleted cells compared to those of the Ti controls (Figure 6). This suggests that the Ag^+^-ions are stably associated with or are being taken up by the bacteria. It should be noted that the cell pellets for the AgO-treated samples were very small compared to the control, as the number of viable cells was much smaller. Additionally, while there was some variability between samples, all bacteria tested exhibited approximately the same amount of Ag associated.

### 2.5. Mammalian Cell Viability

**Figure 7:**
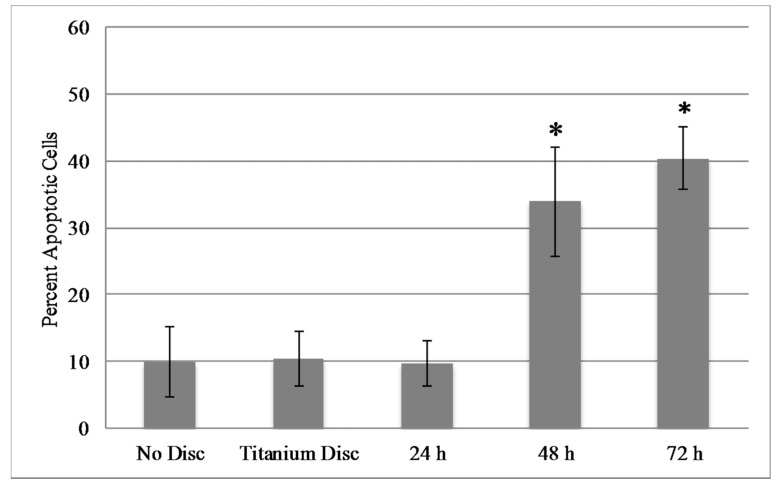
Mammalian in vitro toxicity assays. NIH3T3 (1.5 × 10^6^ cells) were plated and cultured for approximately 12 h. Discs were then added for the indicated time points. Cells were collected and stained with Annexin-V and PI and flow cytometric analysis was performed. Assays were performed in triplicate and the averages are graphed with standard deviations. * *p*-value < 0.05.

Ag^+^ ions have been shown to be toxic to mammalian cells at certain concentrations [22]. Therefore, it was of interest to determine whether Ag*_x_*O discs and the eluted Ag^+^ from these discs are toxic to murine fibroblasts (NIH3T3 cells) in vitro. Cells (1.5 × 10^5^) were seeded in six-well plates in complete media. Cells were incubated with either no disc, uncoated Ti disc, or Ag*_x_*O-coated Ti discs for the indicated times, and apoptosis was analyzed with the Annexin-V assay followed by flow cytometric analysis. Figure 7 shows that apoptosis significantly increased by 48 h in cells incubated with discs coated with 150 nm films on one or both sides of the disc. Interestingly, the percentage of apoptotic cells appears to plateau at approximately 40% in both cases. Of note, it was observed microscopically that cells immediately surrounding the disc showed signs of cell stress prior to cells located distally to the disc, which is likely due to an increased local concentration of Ag^+^ ions after or during elution (data not shown). Collectively, these data suggest that a high silver concentration in the local environment eventually becomes toxic to the local cells.

## 3. Discussion

Reactive magnetron sputtering was shown to produce thin-film coatings containing AgO and/or Ag_2_O that display beneficial materials properties for application to medical and other devices. Overall, the experimental results presented show a distinct and rapid inhibition of bacterial cell growth in planktonic culture and on surfaces due to the presence of Ag*_x_*O coatings. This inhibition is clearly linked to the ability of coatings to elute Ag^+^-ions into solution, or the local environment.

### 3.1. Synthesis of and Analysis of AgO Containing Coatings

The Ag*_x_*O coatings deposited in the work reported here eluted Ag^+^-ions at a rate of approximately 4 × 10^16^ ions/cm^2^ min without a strong dependence on the formed phase, though sample-to-sample runs could vary by a factor of close to 2. The dissolution of Ag-NP was measured by Kent et al. [21]. In this work, pillars of pure Ag are deposited and the pillar profile is imaged/measured with a scanning probe microscope before and after exposure to DI-water or various concentrations of NaCl-containing solutions for specific time intervals. The nanoparticles are approximately 100 nm in diameter and 60 nm tall. Assuming a constant dissolution rate, the pillars dissolve at a rate less than 30 nm^3^/min. If the nanoparticle pillars are assumed to be roughly cylindrical and the initial dimensions of 60 nm tall with a radius of 52 nm are used to calculate the nanoparticle surface area, it is found from the known density of bulk Ag that about 6 × 10^12^ atoms/cm^2^/min are transformed to Ag ions. This Ag ion release was then compared, as shown in Figure 3A, to that for the Ag*_x_*O coatings. These results indicate that the silver oxide coatings, with an elution rate of approximately 2.6 × 10^16^ ions/cm^2^/min, elute Ag^+^-ions at a rate roughly 4000 times greater than that of Ag nanoparticles.

### 3.2. Antibacterial Activity

As mentioned previously, Ag has been used as a method for sterilization for thousands of years [23]. Notably, in that time, there has been limited development of bacterial resistance to Ag [14]. The lack of resistance development is credited to the ability of Ag to induce a response in a variety of targets in bacterial cells, both membrane and cytoplasmic; multiple bacterial targets also contribute to the broad-spectrum activity [12,24,25] of Ag. In reported cases of increased Ag resistance, the main determinant involves a periplasmic metal-binding protein, a chemiosmotic efflux pump, and an ATPase efflux pump [26,27]. These plasmid-encoded pumps, which actively transfer the Ag^+^ out of the cell, are thought to be a major cause of Ag resistance [26,27]. The development of silver resistance is not widespread, which can be attributed to the broad-spectrum activity and the proposed mechanism of action, which target multiple bacterial components [24]. Although resistance to Ag is a possibility, the rate of development appears to be slower than that of alternative antimicrobial agents, which gives promise to future development of Ag-based antimicrobial therapies in combinatorial and mixed-therapy applications [28]. However, one of the main limitations to using Ag as an antimicrobial agent or in combination approaches has been the limited solubility of pure Ag metal in aqueous solutions [16]. Our previous work and that presented here show a viable strategy for developing Ag-based coatings that circumvent the solubility issue through the use of Ag*_x_*O [29].

### 3.3. Broader Perspectives

Ag nanoparticles have also been commonly used as an efficacious and practical way to coat surfaces; recent advances in nanotechnology have afforded the ability to vary the size of the particles, the physical characteristics of the particles, and the dissolution/elution profile [30]. However, nanoparticle coatings have a distinct set of limitations based on the fabrication methodology, including inherent variability in material composition. The use of nanoparticles has also shown variability in their toxicity profile, due to the variability in material composition [31,32]. Nonetheless, nanoparticles do provide a straightforward route to biologically active Ag, although this route is often confounded by variability in elution rates, the mechanical properties of the nanoparticles, and the ability to adhere the nanoparticles to surfaces.

The majority of recently reported work utilizing Ag^+^ compounds as antimicrobials incorporates the use of nanoparticles; in fact, 30% of total nano-products are based on Ag [33]. Nanoparticles have become a popular alternative material for the creation of antimicrobial coatings on medical devices and bandages. Although copper, zinc, Ti [34], magnesium, gold [35], and alginate [36] have all been tested for activity, Ag nanoparticles have proven the most efficacious against a wide range of bacteria and viruses [30]. When three types of nanoparticles were compared in a growth curve analysis to AgNO_3_ as a positive control, only the highest concentrations of both colloidal and biogenic nanoparticles were able to inhibit growth comparable to the positive control in both Gram positive and Gram negative bacterial species [37]. The concentrations of Ag^+^ in the aforementioned study ranged from 0.47 μg/mL to 0.53 μg/mL, with the lowest of those showing no antimicrobial activity [37]. In comparison, the concentrations in the study presented here ranged from 0.35 μg/mL to 1.12 μg/mL, and showed comparable antimicrobial efficacy for both Gram positive and Gram negative species at 0.52 μg/mL (equivalent to 30 min of release in LB).

Numerous studies have shown that nanoparticle delivery of Ag^+^ is more toxic to mammalian cells than delivery via Ag-coated biomaterials [38,39]. Therefore, novel methods for coating biomaterials with various Ag compounds in order to control Ag^+^ release have been investigated [40]. Numerous in vitro and in vivo studies have recently been performed with various Ag-coated materials, and have demonstrated antibacterial efficacy in the absence of mammalian cell cytotoxicity [41,42,43,44,45,46,47]. Our in vitro cytotoxicity results demonstrate that our coatings show cytotoxicity only in a portion of the cell population at 48 h (Figure 7). A microscopic analysis demonstrated that toxicity was apparent in the cells immediately surrounding the disc and not cells located distally, suggesting that that the local concentration of Ag^+^ is higher than that of the rest of the culture (data not shown). Preliminary in vivo results show that subcutaneous implantation of the Ag*_x_*O-coated Ti discs in mice does not result in a detectable increase in Ag^+^ in blood compared to controls up to 21 days (manuscript in preparation). Collectively, these data suggest that in a “closed system”, such as a tissue culture plate, the accumulation of Ag^+^ ions can become cytotoxic, which was also suspected by another group in their model system [46]. However, in an “open system”, such as an animal, the removal of Ag^+^ from the local environment via circulation can reduce or eliminate cytotoxic effects. Additional in vivo experimentation to investigate the toxicity of these particular coating compositions is warranted.

## 4. Materials and Methods

### 4.1. Generation of Ag-Containing Films

Thin films of cubic silver oxide (AgO) were deposited reactively in a custom-designed two-cathode sputter deposition chamber. Each of the cathodes contains a two-inch diameter silver target with 99.95% purity. The cathodes are in a confocal configuration pointing upward toward a sample holder that is 1″ × 2″. The gas mixture used was 67% argon and 33% oxygen adjusted with two mass flow controllers. Argon was adjusted to a rate of 20 sccm while oxygen flows at a rate of 10 sccm, though other mixtures of argon and oxygen also will work to synthesize bactericidal Ag*_x_*O. The pressure in the chamber during deposition was held at 20 mTorr controlled by a butterfly baffle valve connected to a capacitance manometer. The working distance, the distance from the Ag target to the substrate, was approximately 3.5″.

Each of the cathodes was powered with an MDX-500 DC-power supply applying a power of 25–100 W to each cathode. In the chamber configuration, the deposition rate is approximately 17 nm/min at 25 W and increases roughly linearly with power. X-ray diffraction results indicate that the coatings were a combination of cubic phases of AgO and Ag_2_O. The microstructure of the coatings was measured with a field emission scanning electron microscope (LEO 1530VP).

### 4.2. Bacterial Culturing

Bacteria were streaked onto LB–miller agar (BD-Difco, Franklin Lakes, NJ, USA) plates from strains stored in a frozen library (*E. coli* MG1655, *S. aureus* ATCC: 27660). All streaks were stored in a refrigerator at 4 °C. To prepare overnight cultures, a single colony of each bacterial strain was added to LB broth (BD-Difco) in sterile culture tubes. Tubes were placed in the shaking incubator at 37 °C overnight to allow for sufficient bacterial growth. Following the incubation period, dilutions of the overnight culture were made in fresh LB 1:100.

### 4.3. Bacterial Growth Analysis

Dilutions were grown to an optical density at 600 nm (OD_600_) 0.2–0.3 before being diluted a second time to the indicated experimental range. The optical density of a bacterial solution can be used to estimate the number of bacterial cells in solution. Calculations were performed under the assumption that OD_600_ = 1.0 is equivalent to ~10^8^ CFU/mL for *S. aureus* and ~10^9^ CFU/mL for *E. coli* [33,48,49]. The value for *S. aureus* differs from a standard reference in the analysis of bacterial susceptibility [50], but is more consistent with other published reports [51,52,53,54].

Bacterial growth inhibition was investigated using a modified version of the minimal inhibitory concentration (MIC) assay [55]. The bacteria were grown as described above, and subsequently diluted to between 10^5^ and 10^6^ CFU/mL in fresh LB media. Subsequently, 200 μL of this diluted culture was then added to individual wells of a sterile 96-well plate containing either no disc, a Ti disc, or a Ag*_x_*O-coated Ti disk. The plate was then covered and incubated at 37 °C overnight. After incubation, 150 μL of the culture was transferred to a new, sterile 96-well plate and the OD_600_ was measured with a Spectramax M5 multimode plate reader.

For growth kinetics analysis, the bacteria were added to fresh Mueller–Hinton broth in sterile tubes containing the test piece; the total volume in each tube was 3 mL. All experiments with Ag-coated discs were performed in at least duplicate for each bacterial species tested. OD_600_ measurements were recorded on each of the cultures every 30 min with ultraviolet-visible spectroscopy; between time intervals, tubes were agitated at 220 RPM in a shaking incubator at 37 °C.

The coated or uncoated foils were also used to assess the inhibition of bacterial growth on solid agar plates. Briefly, an agar plate was seeded with enough bacteria to form a confluent lawn on the surface and allowed to incubate for 30 min at 37 °C to allow the bacteria to adhere to the surface. Subsequently, the uncoated or Ag-coated Ti discs were applied to the surface to assess a zone of inhibition (ZOI) of bacterial growth similar to the traditional Bauer–Kirby method [56,57]. The plates were allowed to incubate overnight at 37 °C and the ZOI was measured.

### 4.4. ICP-MS Analysis

All samples were prepared with deionized water, phosphate buffered saline (PBS, 50 mM sodium phosphate, 150 mM NaCl, pH 7), sterile LB broth, or DMEM medium. Medium (10 mL) was added to a sample tube, and test discs were subsequently added to the bottom of each tube. Sample tubes were placed in the shaking incubator at 37 °C for ten-minute intervals. Following the incubation period, the media was removed, and an equal volume of fresh media was added to the sample tube; release experiments were performed over a one-hour period.

In the case of bacterial uptake experiments, cells from 1 mL of culture from each of the tubes used in the growth curve experiment were pelleted for 10 min at 6000 RPM in a Benchmark mini centrifuge. The supernatant from each of these tubes was placed into a new sample tube and was combined with 5% nitric acid. The pellet containing the bacterial cells was discarded. All ICP samples were run against a standard prepared in 5% HNO_3_ containing 1000 ppm AgNO_3_ (Ricca, Arlington, TX, USA) calibrated to fit a linear curve model and contain expected experimental outcome values.

### 4.5. Mammalian Cell Viability Assays

NIH3T3 cells (American Type Tissue Culture, Manassas, VA, USA) were cultured in DMEM with 4.5 g/L glucose, l-glutamine, and sodium pyruvate (10-013-CV; Corning, NY, USA) with 10% fetal bovine serum (FBS) (35-010-CV; Corning, NY, USA) and 1% penicillin–streptomycin (P0781; Sigma-Aldrich, St. Louis, MO, USA). NIH3T3 (1.5 × 10^6^ cells) were plated in a six-well plate approximately 12 h before adding discs and incubated at 37 °C with 5% CO_2_. Titanium only (control) or silver-coated titanium discs were gently placed into the wells at the indicated time points before collection. Media was removed and centrifuged to pellet any non-adherent cells; adherent cells were gently trypsinized and pooled with the non-adherent cells for each sample. Cells were collected and stained with an Annexin-V/PI kit (630109; Clontech, Mountain View, CA, USA), and flow cytometric analysis was performed to determine the percentage of apoptotic cells. Student’s *t*-test was performed to determine statistical significance for the mammalian cell in vitro toxicity assays for each sample compared to an untreated control. A *p*-value < 0.05 was considered statistically significant.

## 5. Conclusions

In this study, silver oxide thin-film coatings were applied to Ti foil to test antimicrobial activity as a proof of concept for medical device applications. The coatings were deposited by a reactive sputtering method that allows the process to be performed at room temperature, making both a large-scale manufacturing process and applicability to alternative substrates viable options for future applications. In addition, the sputtering method can be adjusted to produce variability in coatings including chemical composition and adjustable elution rates. Coatings can be developed and tailored via composition changes or the creation of distinct, multi-layered systems in which each layer of the coating has different chemical compositions and/or elution profiles for Ag^+^. In addition to the layering capability, homogeneous coatings of varied chemical compositions can also be used to modulate elution profiles.

## Supplementary Materials

The following are available online. Figure S1: Histograms from flow cytometric analysis used to generate graphs in Figure 7. Panels A, B and C are the three separate replicates used. Table S1: Elution rate of Ag ions in different media types.

## Abbreviations

The following abbreviations are used in this manuscript:

*S. aureus*	*Staphylococcus aureus*
*E. coli*	*Escherichia coli*
Ag*_x_*O	Mixture of AgO and Ag_2_O
PI	Propidium Iodide
OD_600_	Optical density at 600 nm
ZOI	Zone of inhibition
