# Supplementary material for: Silver Oxide Coatings with High Silver-Ion Elution Rates and Characterization of Bactericidal Activity
Source: Molecules. 2017 Sep 7;22(9):1487. doi: 10.3390/molecules22091487 (PMC6151401; doi:10.3390/molecules22091487)
Supplement: Supplementary file 1 [file molecules-22-01487-s001.pdf]

Silver oxide coatings with high silver-ion elution rates and characterization of bactericidal activity

Sarah S. Goderecci [1,†], Eric Kaiser[2†], Michael Yanakas[2], Zachary Norris[2], Jeffrey Scaturro[2], Robert Oszust[2], Clarence D. Medina[1], Fallon Waechter[1], Min Heon[6], Lei Yu[1], Samuel E. Lofland[2], Renee M. Demarest[4], Robert R. Krchnavek [3], Gregory A. Caputo [1,5*] and Jeffrey D. Hettinger [2,5*]

Supplemental Figures and Tables

A

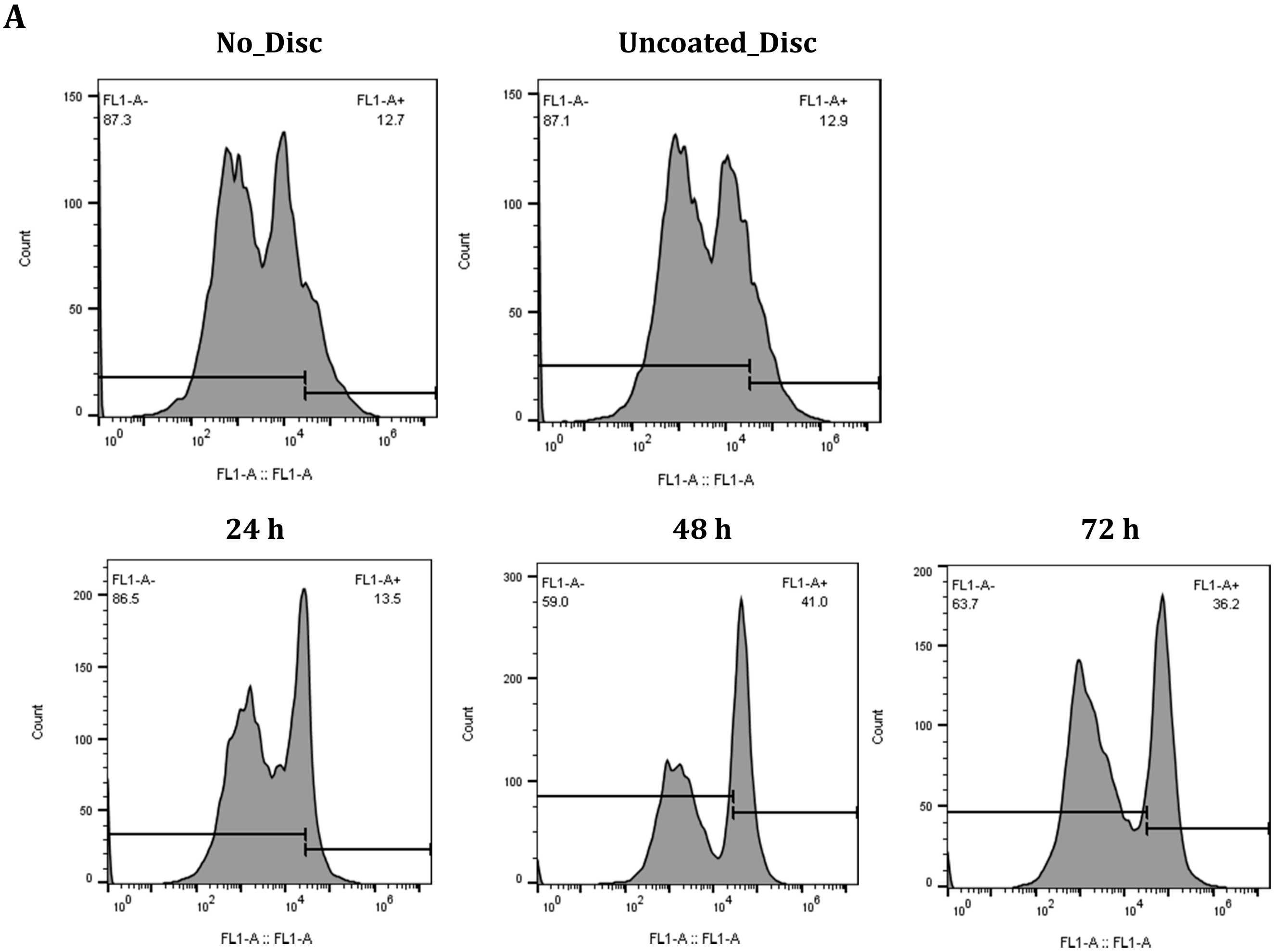

B

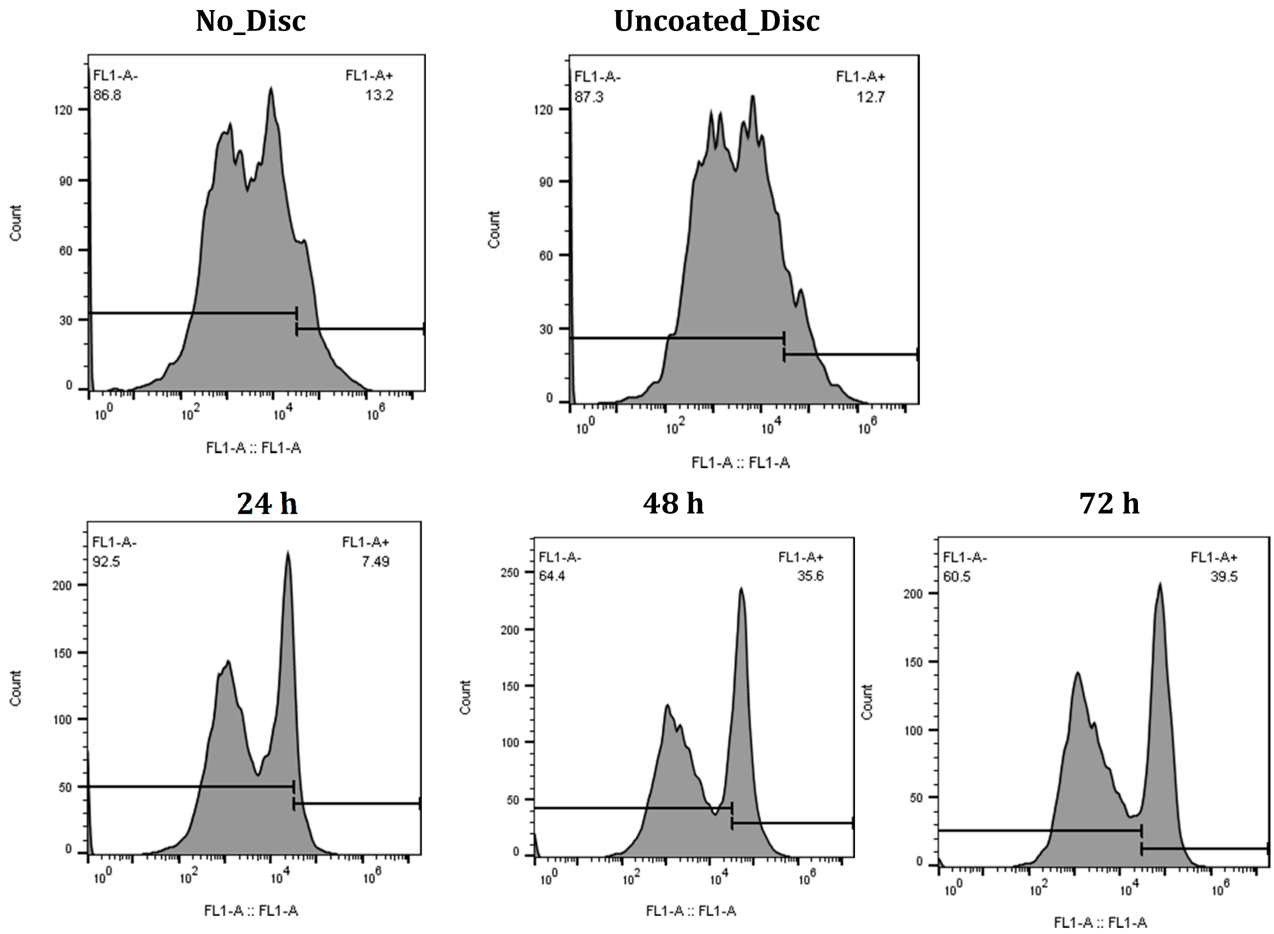
No_Disc
FL1-A-
86.8
FL1-A+
13.2
Count
FL1-A :: FL1-A
Uncoated_Disc
FL1-A-
87.3
FL1-A+
12.7
Count
FL1-A :: FL1-A
24 h
FL1-A-
92.5
FL1-A+
7.49
Count
FL1-A :: FL1-A
48 h
FL1-A-
64.4
FL1-A+
35.6
Count
FL1-A :: FL1-A
72 h
FL1-A-
60.5
FL1-A+
39.5
Count
FL1-A :: FL1-A

C

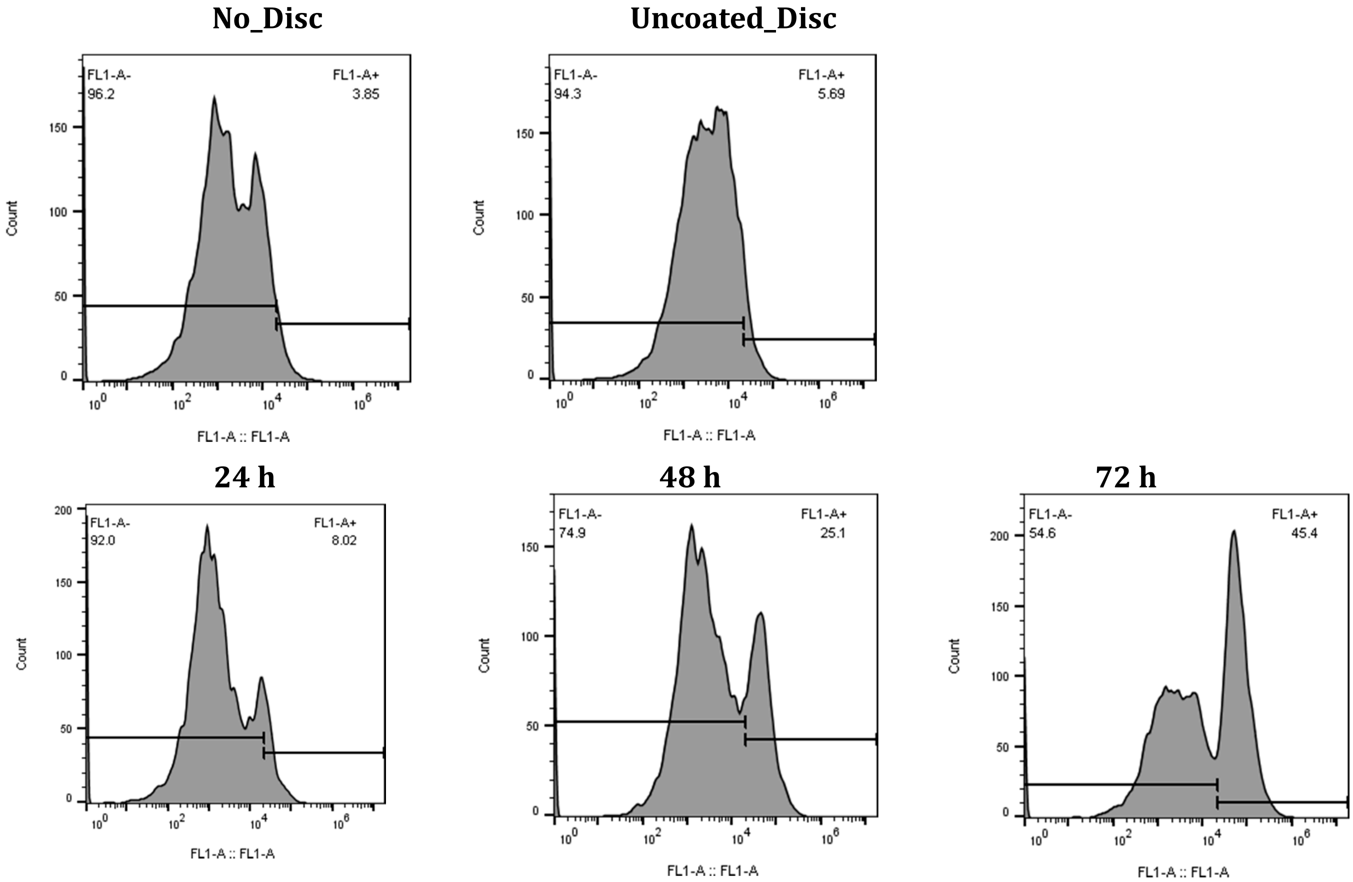
No_Disc
FL1-A-
96.2
FL1-A+
3.85
Count
FL1-A :: FL1-A
Uncoated_Disc
FL1-A-
94.3
FL1-A+
5.69
Count
FL1-A :: FL1-A
24 h
FL1-A-
92.0
FL1-A+
8.02
Count
FL1-A :: FL1-A
48 h
FL1-A-
74.9
FL1-A+
25.1
Count
FL1-A :: FL1-A
72 h
FL1-A-
54.6
FL1-A+
45.4
Count
FL1-A :: FL1-A

Supplemental Figure 1 - Histograms from flow cytometric analysis used to generate graphs in Figure 7. Panels A, B, and C are the three separate replicates used.

Supplemental Table 1

Table S1			
Elution rate of Ag ions in different media types			
Media Type	Elution Rate (ppm/min)[a]		R^2 Value[a]
Water	7.55E-02	± 0.0071	0.998
PBS	3.34E-02	± 0.0058	1.000
LB Broth	4.55E-03	± 0.0011	0.995
DMEM	3.65E-03	± 0.0006	0.996

a – Rates and R^2 values presented are the average of the rates R^2 values of each individual replicate of elution experiments (3-6 replicates).
